# Concomitant endocarditis and spondylodiscitis due to coagulase-negative Staphylococci and a review of the literature

**DOI:** 10.1016/j.idcr.2021.e01100

**Published:** 2021-03-31

**Authors:** Karlijn M.G. Houkes, Saskia E. Mudde, Alina A. Constantinescu, Nelianne J. Verkaik, Erlangga Yusuf

**Affiliations:** a Department of Medical Microbiology and Infectious Diseases, Erasmus University Medical Center, Rotterdam, the Netherlands; b Department of Cardiology, Thorax Center, Erasmus University Medical Center, Rotterdam, the Netherlands

**Keywords:** Endocarditis, Spondylodiscitis, Coagulase-negative staphylococci

## Abstract

**Background:**

Coagulase-negative staphylococci (CoNS) are part of the normal skin flora. Although CoNS are generally considered as low pathogenic microorganisms, they can cause serious infections, particularly in the context of foreign body material.

**Case report:**

Here we present two cases of concomitant infectious endocarditis and spondylodiscitis; one caused by *S. epidermidis*, the other by *S. haemolyticus*. Additionally, we reviewed the literature for previously reported cases of concomitant endocarditis and spondylodiscitis due to CoNS.

**Conclusion:**

In patients with back pain and a cardiac device in situ, CoNS should be considered as causative pathogens for possible endocarditis and/or spondylodiscitis, and should not be regarded as contamination.

## Highlights

• When CoNS are found repetitively in the context of cardiac foreign body materials, endocarditis should be considered.
• Given the subacute nature of CoNS-infections, one needs to alert to endocarditis, even if the symptoms are still mild.
• When CoNS-related endocarditis patients complain of back pain, concominantly occuring spondylodiscitis should be considered.
• Concominant endocarditis and spondylodiscitis caused by CoNS is rare with only 12 cases reported in literature so far.

## Background

Coagulase-negative staphylococci (CoNS) are colonizers of the human skin and are generally considered to be low pathogenic [1]. They are particularly associated with low-grade infections related to foreign-bodies, such as intravascular catheters, prosthetic joints or cardiac devices [1]. However, more severe and less prevalent infections, such as endocarditis and spondylodiscitis, have also been linked to CoNS. It is estimated that approximately 10 % of cases of infective endocarditis are caused by CoNS [2]. Similar proportions have been reported for CoNS-related spondylodiscitis [3]. Here, we present two cases with concomitant endocarditis and spondylodiscitis attributed to CoNS and provide a review of the literature.

## Cases

### Patient A

**Fig. 1:**
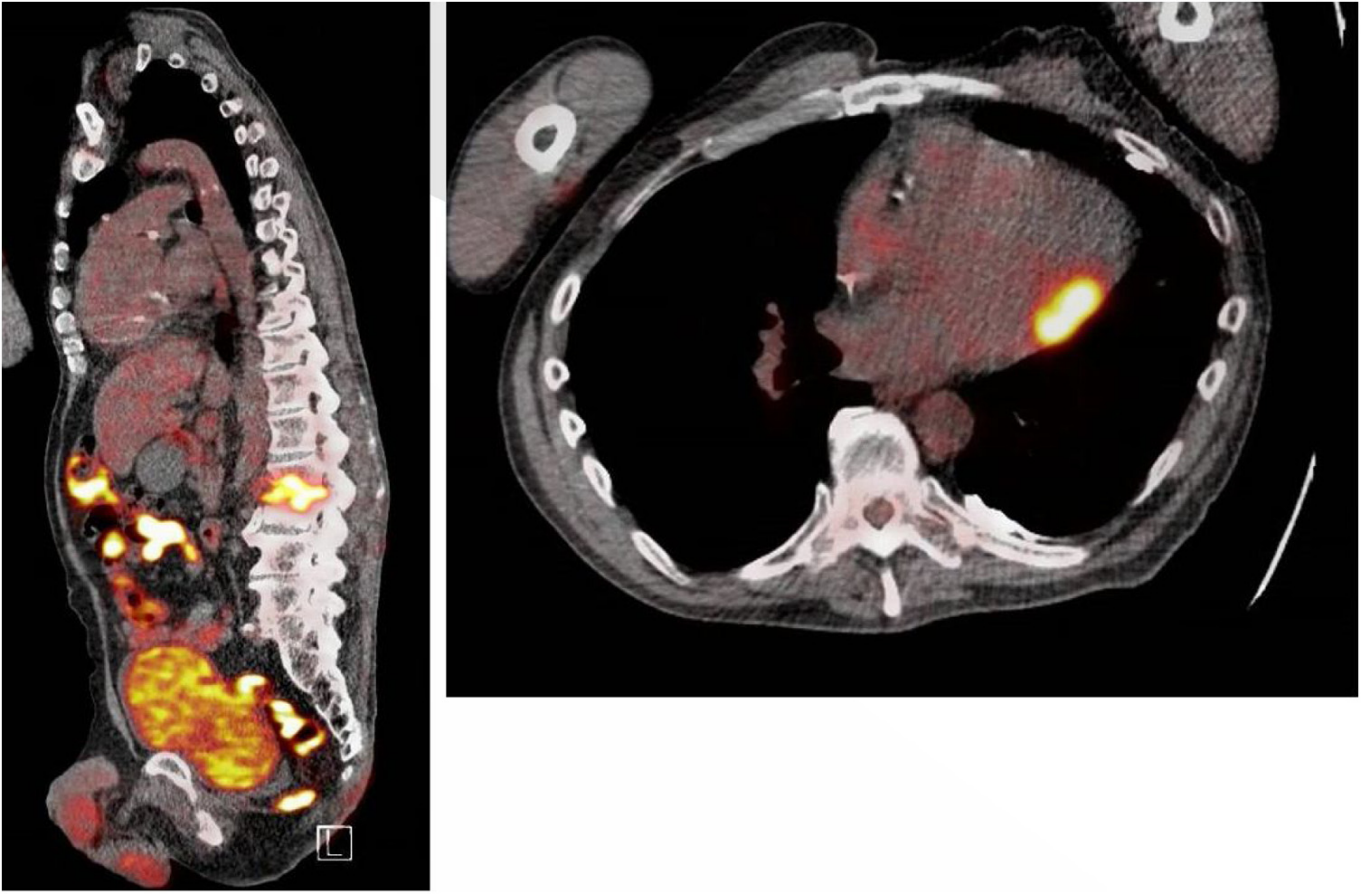
PET-CT scan of patient A showing increased FDG-uptake at lumbar discus L2-L3 (left panel) and in the heart (right panel).

A 65-year-old man with diabetes mellitus type 2 presented to our hospital (Erasmus Medical Center, Rotterdam, The Netherlands) after he was found unconscious with hypoglycemia. His medical history included a non-invasive urothelial carcinoma and chronic heart failure. For the latter, he received an Implantable Cardioverter-defibrillator (ICD) 12 years ago. It was replaced three months before his current admission to our hospital, because the leads were visible through a local inflamed skin defect. Since then, he had lost 8 kg in body weight, and during the past three weeks he complained of progressive lower back pain. On presentation, his white blood cell count was 13.8 × 10^9^/l (normal value: 3.5–10 × 10^9^/l) and C-reactive protein 58 mg/l (normal value: <10 mg/l). A CT-scan of the abdomen showed lesions suggestive of spondylodiscitis at the level of L2 and L3 with epidural involvement. All four blood culture sets taken shortly after hospital admission remained negative after 5 days of incubation. After CT-guided lumbar biopsies were obtained, empirical treatment was started with cefuroxime 1500 mg i.v., thrice daily. Tissue cultures grew methicillin-resistant *S. epidermidis,* which was initially considered to be a contaminant. To evaluate possible sources of the spondylodiscitis, a PET-CT scan was performed (Fig. 1). FDG-avid lesions were seen at L2 and L3, and in the heart at the lateral wall of the left ventricle. On a subsequent transthoracic echocardiogram (TTE), a mobile structure attached to the ICD lead and the tricuspid valve was observed. Culture-negative, ICD-related endocarditis was suspected, and the ICD was extracted the following day. Instead of continuing cefuroxime, the therapy was switched to ceftriaxone 2000 mg once daily and vancomycin 1000 mg i.v. twice daily (adjusted to his weight), according to national antibiotic guidelines (SWAB) [4]. Four days later, progression of the lumbar lesions was seen on a follow-up MRI scan, despite antimicrobial therapy. The scan revealed abscess formation in the discus between L2 and L3 with multiple abscess pockets in the adjacent psoas muscle. There was no extensive epidural expansion of the abscess. The C-reactive protein had declined to a value of 24 mg/L. Since his symptoms did not deteriorate, the patient was treated conservatively. When blood cultures that were taken on consecutive days after ICD-extraction became positive for *S. epidermidis* with identical susceptibility patterns, this was considered to be the causative pathogen of both endocarditis and spondylodiscitis. Ceftriaxone was stopped and vancomycin was continued for 6 weeks following the last positive blood culture. The patient was discharged in good condition after three months of hospital admission. No control MRI scan was performed.

### Patient B

**Fig. 2:**
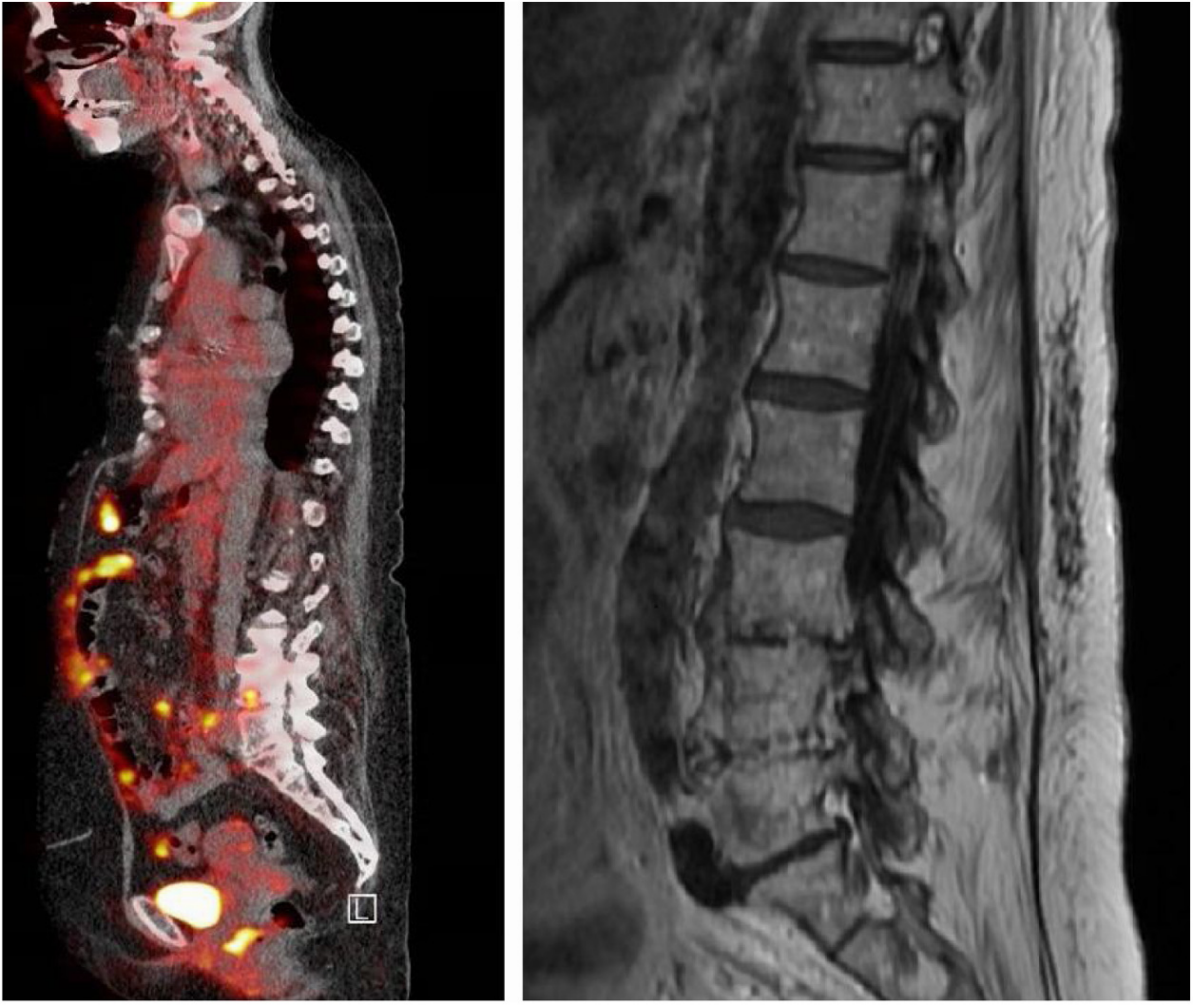
PET-CT scan (left panel) and MRI scan (right panel) of patient B revealing increased FDG-uptake at lumbar discus L4-L5, indicative for spondylodiscitis.

A 66-year-old woman presented to our hospital with complaints of fever, malaise and lower back pain in the last weeks. Her medical history included auto immune hepatitis, for which she used prednisone and azathioprine; Hodgkin lymphoma; transcatheter aortic valve implantation (TAVI) due to aortic stenosis; and two episodes of suspected TAVI-related endocarditis, caused by *E. faecalis* and *E. faecalis* and *S. haemolyticus*, respectively, with a 7 month-interval. The first episode was treated with amoxicillin 2 g i.v., six times a day, and ceftriaxone 2 g, twice daily, for 6 weeks according to the SWAB guidelines [4]. The second episode was treated with amoxicillin 2 g, i.v., six times a day, and daptomycin 10 mg/kg/day for a total of 6 weeks with a good clinical response. Initially she received vancomycin for 5 days, but this was switched to daptomycin when the patient developed tubulointerstitial nephritis possibly due to vancomycin [4]. During both episodes, several imaging tests were performed, but none of them showed vegetations on the heart valves. The present complaints started 4.5 months after the second episode. Laboratory findings showed C-reactive protein of 112 mg/l and a white blood cell count of 13.3 × 10^9^/L. One blood culture bottle obtained at the emergency department grew methicillin-resistant *S. haemolyticus* (daptomycin MIC 0.25 μg/mL, vancomycin MIC 1 μg/mL). Since this organism was also found during the second episode of suspected endocarditis, the patient was admitted. The additional blood cultures grew *S. haemolyticus* as well. Because a third episode of endocarditis was suspected, a TTE and PET-CT-scan were performed. The TTE showed a mobile structure on the prosthetic aortic valve. Although no signs of endocarditis were seen on the PET-CT scan, the scan did reveal spondylodiscitis of discus level L4-L5, which was confirmed on a MRI scan performed on the same day (Fig. 2). Due to extensive comorbidity, including radiotherapy for Hodgkin lymphoma, chirurgical intervention was not possible. No tissue biopsies were obtained. Intravenous daptomycin (10 mg/kg) was intended to be given for 12 weeks, but the patient deceased suddenly in the 5th week of antibiotic treatment. After initiation of antimicrobial therapy, the blood cultures remained positive for *S. haemolyticus* for three more days. No obduction was performed and the exact cause of death remained unknown.

## Discussion

We presented two cases of concomitant endocarditis and spondylodiscitis due to CoNS. CoNS cause approximately 10 % of cases of infective endocarditis [2] as well as about 10 % of the spondylodiscitis cases [3]. However, only 12 cases of concomitant endocarditis and spondylodiscitis have been reported in literature (Table 1). Among the reported cases of concomitant endocarditis and spondylodiscitis, *S. epidermidis* was identified in half of the published cases. Two cases were caused by *S. lugdunensis* (Table 1), but this microorganism, despite being categorized as a CoNS, is more virulent than other CoNS and closely resembles *S. aureus* in this aspect [5]. Interestingly, none of the previously reported cases were due to *S. haemolyticus*, which makes patient B the first described case of *S. haemolyticus*-related concomitant endocarditis and spondylodiscitis.

**Table 1:** Clinical features of previously reported cases of concomitant endocarditis and spondylodiscitis caused by coagulase negative staphylococci.

Case	Age/sex	Predisposing condition	Endocarditis		Spondylodiscitis		Causative organism	Antibiotic treatment	Treatment duration	Outcome	Reference
			Location (heart valve)	Diagnosed by	Location (vertebrae)	Diagnosed by					
1	74/F		MV	Blood cultures (repeatedly), autopsy	T7	Bone scintigraphy, autopsy	*S. epidermidis*	PEN, GEN, LEX, ERY, CXA	6 weeks	Died	[19]
2	66/M	Colon resection for villous adenoma, right total hip replacement	AV, MV	Echocardiogram, blood cultures (multiple)	L2-L3	Radiography, bone scintigraphy, tissue culture	*S. warneri*	VAN, GEN, RIF, DCX	6 weeks	Survived	[20]
3	64/M	AV replacement for aortic regurgitation	AV	Blood cultures	L2-L3	Radiography, bone scintigraphy	*S. epidermidis*	PEN G, GEN, FA, TEC, FLX	5 months	Survived	[21]
4	65/M	Submucosal resection of urothelial carcinoma	MV	Blood cultures	T10	Radiography, bone scintigraphy	*S. epidermidis*	PEN, GEN, FLX	3.5 months	Survived	[22]
5	78/M	Pacemaker	TV	Blood cultures (multiple), cultures of pacemaker electrode	L4-L5	CT-scan, chronic osteomyelitis on histologic examination.	*CoNS*	FLX, NET, VAN, RIF, TEC, CLI	At least 17 weeks	Survived	[23]
6	77/F	Chronic Lymphatic Leukemia (with central venous access device)		Blood cultures (multiple), cultures of catheter tip	T11-T12	Radiography, MRI, positive tissue culture	*CoNS*	PEN G, CIP, RIF	12 weeks	Survived	[23]
7	68/M	Coronary artery disease, sick sinus syndrome, pacemaker		Blood cultures (multiple)	L3-L4	Radiography, CT-scan, chronic inflammation on histologic examination.	*S. lugdunensis*	PEN G, GEN, FLX, RIF, CIP		Survived	[23]
8	79/M	Psoriasis, rheumatoid arthritis (corticosteroids, methotrexate), left total knee replacement	AV. MV	Blood cultures (repeatedly), TEE, TTE, autopsy		MRI scan, autopsy	*S. lugdunensis*	RIF, CIP, CLI, VAN	5 months	Died	[24]
9	82/M	Pacemaker		Duke criteria	C2-C3	One of the following: radiography, bone scintigraphy, CT-scan or MRI scan	*S. schleifferi*	VAN, RIF			[16]
10	72/M	Pacemaker		Duke criteria	L2-L3	One of the following: radiography, bone scintigraphy, CT-scan or MRI scan	*S. epidermidis*	OXA, RIF			[16]
11	78/M	Pacemaker		Duke criteria	T8-T9	One of the following: radiography, bone scintigraphy, CT-scan or MRI scan	*S. epidermidis*	OXA, RIF			[16]
12	64/M	Pacemaker		Duke criteria	L1-L2	One of the following: radiography, bone scintigraphy, CT-scan or MRI scan	*S. epidermidis*	OXA, GEN			[16]

AI = aortic insufficiency; AS = aortic stenosis; ICD = implantable cardioverter defibrillator; MV = mitral valve; AV = aortic valve; TV = tricuspid valve; CoNS = coagulase negative staphylococci; PEN = penicillin; GEN = gentamicin; LEX = cephalexin; ERY = erythromycin; CXA = cloxacillin; NAF = nafcillin; VAN = vancomycin; RIF = rifampicin; DCX = dicloxacillin; PEN G = penicillin G; FA = fusidic acid; TEC = teicoplanin; FLX = flucloxacillin; NET = netilmicin; CLI = clindamycin; CIP = ciprofloxacin; OXA = oxacillin; NET = netilmicin.

It can be challenging to determine the relevance of an isolated CoNS, especially if only a single blood- or tissue culture is positive. In both patient A and B, *S. epidermidis* and *S. haemolyticus*, respectively, were at first considered to be a contaminant. Only after additional blood cultures were positive for these microorganisms, they were considered to be the causative microorganisms. CoNS as pathogens in native valve endocarditis are rarely found [6]. However, in prosthetic valve endocarditis, CoNS are the second leading cause, accounting for 20.1 % of the cases [7]. Additionally, six out of 12 described cases of concomitant endocarditis and spondylodiscitis had a pacemaker. Together, this data indicates that the presence of foreign body material in the heart is a risk factor for developing CoNS-related endocarditis. This is supported by a finding by Chu et al., that showed that significantly more patients with native valve endocarditis caused by CoNS had a cardiac device, compared to patients with *S. aureus*- or viridans group streptococci-related endocarditis [6]. Interestingly, both patient A and patient B had undergone surgery in or near the heart (ICD replacement and TAVI, respectively) in the year prior to the endocarditis episode. The reported incidence of infectious endocarditis after aortic valve replacement is 0.9 % in the first year after surgery [8], in which CoNS-related endocarditis is 37 % [9]. The proportion of CoNS-related endocarditis declines to 18 % after the first year following replacement (p = 0.005) [9]. Cardiac device-related endocarditis prevalence has been reported between 0.5–7% [10]. In patients with cardiac device-related endocarditis, staphylococci (predominantly CoNS) account for 60–80 % of the cases [11]. CoNS can originate from the skin flora of the patient or from health care personnel during surgery and can enter into the human body during insertion of a medical device [12]. Subsequently, CoNS could thrive there by forming a biofilm on the foreign body surface of for example artificial valves, pacemakers or ICDs (2). Presumably, the ICD of patient A and the prosthetic aortic valve of patient B have attributed to the development of CoNS-related endocarditis.

The incidence of spondylodiscitis as a complication of endocarditis varies widely, ranging from 0.02 % [15] to up to 15 % [16]. This variability could partially be explained by the virulence of the involved pathogens. Le Moal et al. reported CoNS as the most common pathogens among their tested patient population, whereas viridans group streptococci and *S. aureus* were best represented in the study performed by Gonzalez-Juanatey et al. [15,16]. Given that endocarditis due to CoNS is often chronic with a subacute presentation, lacking the characteristic symptoms of a severe infection caused by for example *S. aureus* [17], we hypothesize that long-lasting CoNS bacteremia as a consequence of delay in the diagnosis of subacute infectious endocarditis may lead to prolonged exposure of bone tissue to CoNS. This might result in a higher risk of developing spondylodiscitis. However, this could also account for a subacute presentation of CoNS-related spondylodiscitis causing secondary endocarditis. In both cases, it remains unclear whether the endocarditis or the spondylodiscitis was the initial infection. However, we deemed it most likely that the infections started with the endocarditis, since patient A had a recently replaced ICD and patient B had a recently placed prosthetic valve.

Diagnosing spondylodiscitis can be difficult, since back pain is a common complaint and, even in the case of spondylodiscitis, it is not always the main complaint. Likewise, back pain was no dominant symptom for patient B. The suspected spondylodiscitis was only found in the screening for metastatic infections secondary to the infectious endocarditis and confirmed on a subsequent MRI scan. Of the reported cases in literature with concomitant endocarditis and spondylodiscitis, at least 6 did not undergo a MRI scan or PET-CT scan, even though a MRI scan is considered to be the best way to confirm suspected spondylodiscitis [18]. In case of patient B, back pain had been reported by the patient upon admission. Therefore, we advise to actively ask patients with CoNS-related endocarditis if they have back pain and to consider imaging following an affirmative answer. In patient A, lower back pain was a major complaint and spondylodiscitis was identified quickly on the PET-CT scan. However, the PET-CT scan was not suspected of endocarditis, while clear vegetations on the ICD leads and tricuspid valve were seen on the TTE. The presence of a cardiac device is a risk factor of CoNS infections [1]. The IDSA guidelines for treatment and diagnosis of native vertebral osteomyelitis states that efforts should be made to exclude contamination when a common skin contaminant, eg CoNS, is isolated [18]. In patients with sustained CoNS bloodstream infection and an intravascular device or prosthetic valve, we propose to exclude endocarditis by performing a TEE or PET, respectively. In case of endocarditis, extraction of the cardiac device is indicated to control the infection, whenever possible. The ICD of patient A was safely extracted, but the TAVI of patient B could not be replaced as cardiac surgery was not possible.

## Conclusion

We described two cases of concomitant endocarditis and spondylodiscitis due to CoNS, *S. epidermidis* and *S. haemolyticus*. As the numbers of patients with foreign body materials are rising, it is likely that CoNS will become increasingly significant pathogens [1]. Especially when CoNS are repetitively found in the setting of prosthetic valves or cardiac devices, endocarditis should be considered in an early stage, even if symptoms are (still) mild given the subacute nature of CoNS-infections [17]. These cases also illustrate that CoNS are capable of causing spondylodiscitis, which should be considered when patients report back pain and cultures are positive with CoNS. This way, missing such important diagnoses can be prevented and correct antibiotic treatment can be administered.

## Funding

This research did not receive any specific grant from funding agencies in the public, commercial, or not-for-profit sectors.

## Ethical approval

Not applicable.

## Informed consent

Written informed consent was obtained from the patient for publication of this case report and accompanying images. A copy of the written consent is available for review by the Editor-in-Chief of this journal on request.

## Author statement

We confirm that the manuscript has been read and approved by all named authors and that there are no other persons who satisfied the criteria for authorship but are not listed. We further confirm that the order of authors listed in the manuscript has been approved by all of us.

## Author contributions

**Karlijn M.G. Houkes**: Conceptualization, Data curation, Investigation, Writing – Original draft. **Saskia E. Mudde**: Conceptualization, Data curation, Investigation, Writing – Original draft, Visualization. **Alina A. Constantinescu**: Resources, Writing - Review & Editing. **Nelianne J. Verkaik**: Conceptualization, Resources, Writing – Review & Editing. **Erlangga Yusuf**: Conceptualization, Writing – Review & Editing, Supervision.

## Declaration of Competing Interest

The authors report no declarations of interest.
